# Air Leakages at Microvalves: Pressure Decay Measurements and Extended Continuum Modelling of Knudsen Flows

**DOI:** 10.3390/mi15101263

**Published:** 2024-10-16

**Authors:** Daniel Anheuer, Johannes Schwarz, Patrick Debera, Klaus Heinrich, Christoph Kutter, Martin Richter

**Affiliations:** Fraunhofer Institute for Electronic Microsystems and Solid State Technologies, 80686 Munich, Germany; johannes.schwarz@emft.fraunhofer.de (J.S.); martin.richter@emft.fraunhofer.de (M.R.)

**Keywords:** silicon, microvalve, leakage, pressure decay, experimental characterization, simulation, flap valves, OpenFOAM, measurement

## Abstract

To improve the performance of valves in relation to the leakage rate, a comprehensive evaluation of the valve characteristics and behavior during pressure exposure is important. Often, these low gas flow rates below 0.1 cm^3^/min cannot be accurately measured with conventional flow sensors. This paper presents a small and low-cost test rig for measuring gas leakage rates accurately, even far below 0.1 cm^3^/min, with the pressure decay method. These leakage flows are substantiated with a flow model, where we demonstrate the feasibility of modeling those gas flows with an extended Navier–Stokes framework to obtain more accurate theoretical predictions. As expected, the comparison to the experimental results proves that the classical Navier–Stokes system is unsuitable for modeling Knudsen flows. Hence, self-diffusion of gas, a wall-slip boundary condition, and an effective mean free path model were introduced in a physically evident manner. In terms of the calculated mass flow, while self-diffusion and slip boundary conditions explain deviations from the classical Navier–Stokes equation for Knudsen numbers already smaller than 1, the effective mean free path model has an effect, especially when *Kn* > 1. For simplified conditions, an analytical solution was presented and compared to the results of an OpenFOAM CFD-solver for flow rates through more complex gap-flow geometries of the flap valve. Hereby, acceptable deviations between 10% and 20% were observed. A comparison with measurement results was carried out. The reproducibility of the measurement method was verified by comparing multiple measurements of one silicon microvalve sample to a state-of-the-art flow sensor. Three geometrically similar passive silicon microvalves were measured with air overpressure decreasing from 15 kPa relative to atmospheric pressure. Maximum gas volume flowing in a blocking direction of 1–26 µL/min with high reproducibility and marginal noise were observed.

## 1. Introduction

Microvalves as components of micropumps have been developed, improved, and used in different areas and fields of industrial and medical technology [1,2]. Reliable active or passive microvalves as standalone components enable a variety of different microfluidic systems and applications by regulating or switching fluid flows [1]. Fraunhofer EMFT develops and manufactures silicon micro-diaphragm pumps with piezoelectric actuators and passive flap valves [3]. To generate and maintain high air pressure differences with micropumps and passive flap valves is particularly challenging and relevant for use in applications such as vacuum micropumps [4] and micro gas compressors [5]. The valve leakage rate therefore plays a significant role in this context and can be defined as an undesired fluid flow against the pumping direction. For micropumps, the leakage rate reduces important parameters, such as flow rate and back pressure capability [6], which impairs the performance of the pump. In the case of micro-diaphragm pumps with passive flap valves, this is especially important since the actuator movement generates alternating positive and negative pressure. This pressure is applied during each cycle to the closed valve, causing undesired leakage rates.

Small leakage rates, which are significantly lower compared to the transient volume flow of a pump stroke, have no significant effect on the delivery rate of the micropump without back pressure. For this reason, leakage rates of check valves have not yet been the focus of scientific research. However, these leakage rates can lead to backflow in the event of counterpressure, reducing the back pressure performance. Additionally, while the micropump is not in operation, this distorts the dosage amount. When dosing medication, that leads to a backflow of body fluid into the catheter, potentially causing occlusion. In addition, completely new applications such as micro-vacuum pumps can only be realized with check valves with an extremely low leakage rate. As micropumps are increasingly used in corresponding applications, the issue of the leakage rate of passive check valves is becoming more of a focus. On the one hand, it is necessary to understand the influencing factors on which leakage rates depend and to design the check valves accordingly, and on the other hand, the metrology must be developed to measure these leakage rates.

Measuring leakages for micropumps or microvalves is typically challenging with known characterization setups, such as mass flow sensors, due to the very small flow rates involved, especially for gases. In this study, a cost-effective and simple measurement setup is presented, which allows the reliable measurement of smallest gas flow rates below 0.1 cm^3^/min with the pressure decay method. In particular, two low-cost differential pressure sensors with different pressure measuring ranges are used for the precise measurement procedure and compared with each other. Although only one pressure sensor is necessary for this measuring method, we want to demonstrate that by selecting pressure sensors with different measuring ranges, this method can be adapted to the expected areas of interest. Exemplary results of leakage rate measurements are shown using multiple silicon microvalves with similar geometries. Additionally, one silicon microvalve is measured multiple times with the pressure decay method and compared to a reference mass flow sensor. Furthermore, that measurement methodology is substantiated with an extended Navier–Stokes model that includes the use of a modified OpenFOAM v2006 CFD-Solver [7] for the corresponding set of conservation equations in order to quantify the microvalves’ leakage gas flows through gaps, whose geometry is given by static-mechanical finite element method (FEM) simulations, where gap-heights in the sub-micrometer range were obtained. As shown later, these are characterized by high Knudsen numbers (Kn>0.01) and can therefore not be accurately predicted by the classical continuum method based on the conventional Navier–Stokes equations.

## 2. Materials and Methods

This chapter provides an overview of silicon micropumps and silicon microvalves with respect to their fabrication and geometry. A static mechanical simulation in Ansys for the calculation of the remaining gap geometry in case the flap is pressed on the valve seat is presented. Possible causes of leakage rates in those devices are detailed, and multiple measurement methods are described.

### 2.1. Silicon Microvalves and Micropumps

**Figure 1:**
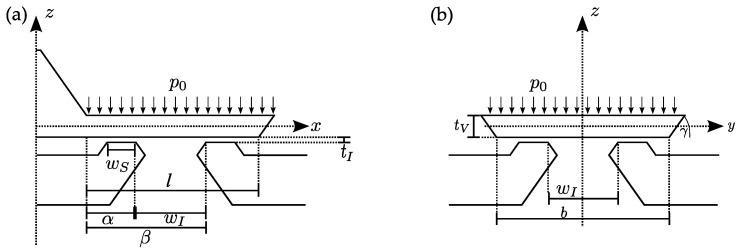
Schematic cross-section of a normally closed passive flap silicon microvalve with the relevant design parameters. Arrows indicate the acting pressure $p_{0}$ in blocking direction of the flap is responsible for the resulting leakage rate. Cross-section along x-z plane through passive flap valve and inlet opening (**a**) and through the center of the valve inlet opening and seating along the y-z plane (**b**).

Figure 1 depicts the schematics of the simulated and characterized silicon microvalve with the relevant dimensions and the acting pressure $p_{0}$ responsible for the leakage rate. A cross-section along the x-z plane illustrates the following relevant geometrical parameters: flap length l, valve seat width $w_{S}$, length from valve clamping to start of inlet opening α, opening length $w_{I}$, length from valve clamping to end of inlet opening β, and opening pressure $p_{0}$ (Figure 1a). The cross-sectional view along the y-z plane through the valve and inlet opening center depicts additional parameters: valve thickness $t_{v}$, valve width b, characteristic KOH etch angle γ, opening width $w_{I}$, and initial gap $t_{I}$ (Figure 1b). Several of the parameters shown are defined according to the lithography mask design or the material and process properties of the numerous manufacturing steps.

**Figure 2:**
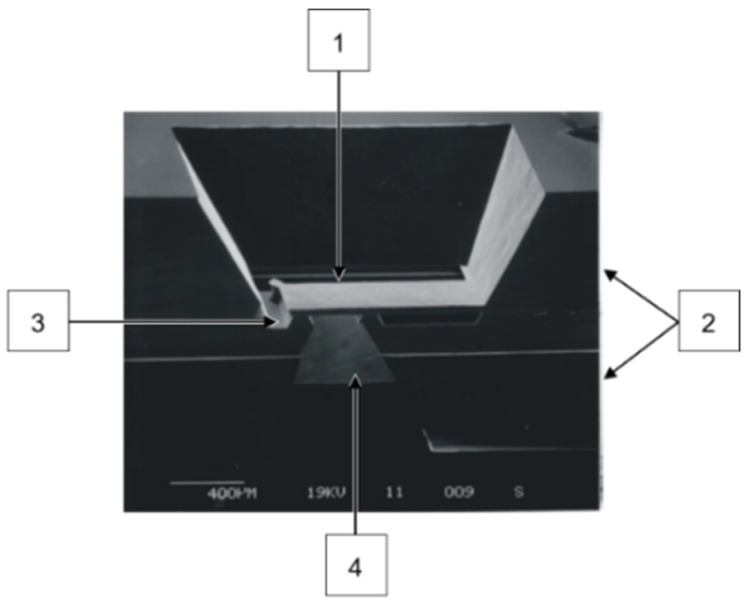
SEM image of silicon microvalve with valve flap (1), both silicon substrates (2), valve seat (3), and inlet opening (4). The valve seat is etched from the front side; the inlet opening is etched from the backside through the wafer.

**Figure 3:**
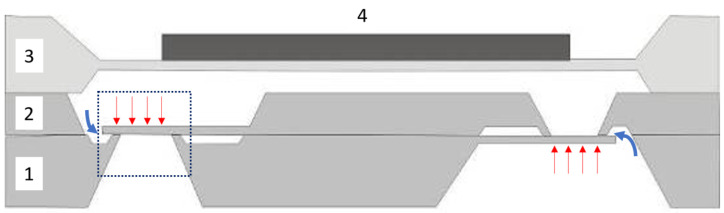
Schematic cross-section of a piezoelectrically driven silicon micropump. (1) Lower valve wafer, (2) thinned upper valve wafer, (3) pump chamber wafer, and (4) piezoelectric actuator. Blue arrows show leakage flow around silicon microvalves; red arrows indicate the applied closing pressure due to the reoccurring pumping cycle caused by the piezoelectric actuator. Blue dotted area is shown in Figure 2.

A scanning electron microscope (SEM) image of a silicon microvalve is shown in Figure 2 with the valve flap (1), the bonded silicon wafers (2), the valve seat (3), and the inlet opening (4). Figure 3 illustrates the schematic cross-section of a silicon micropump developed and manufactured by Fraunhofer EMFT (Munich, Germany) [3]. It consists of two identical valve wafers that are inverted and bonded to each other by a silicon–dioxide direct bond. On the bottom side of substrate 3, the cylindrical pump chamber is etched into the silicon, and the residual membrane is thinned to a thickness of 30–100 µm, depending on the required pump parameters. On the top side of substrate 3 is the glued-on piezoelectric actuator.

The operating principle of this diaphragm pump is based on the inverse piezoelectric effect. As a result of an electrical actuation signal, the piezoelectric ceramic expands and deflects the silicon diaphragm upwards, thus increasing the volume in the pump chamber. The resulting underpressure opens the inlet valve, and fluid flows in the pump chamber. Reversing the actuation voltage causes the piezoelectric actuator to move down toward the valve wafer, causing an overpressure in the pump chamber, which opens the outlet valve and pushes the fluid out of the chamber, and therewith completes the pump cycle. Only the passive silicon flap valve in blocking direction as part of a silicon micropump or as a standalone component is relevant in this publication. Table 1 depicts the designed geometric parameters used for the simulation and fabrication, which are also graphically shown in Figure 1.

### 2.2. Leakage Occurance in Silicon Microvalves

A leakage rate is the undesired flow of fluid against the flow direction of the microvalve. The fluid flows past the valve flap and the valve seat and reduces the achievable flow rate as well as the ability to generate high pressures.

For this study, passive silicon check valves were selected for the following reasons:

- The two surfaces (valve flap and valve seat) that come into contact when the valve closes are very smooth and uniform. The roughness is determined by the CMP-polished surfaces during wafer production and is approximately 0.2…0.4 nm. With a hard-hard contact between valve flap and valve seat, the leakage is negligible. In comparison, other materials exhibit a significantly higher level of roughness, with values reaching several micrometers for plastics and ~100 nm for metals. This highlights the superior performance of silicon valves with hard-hard contact in terms of remaining roughness gaps, provided that no further gaps are created.
- Monocrystalline silicon is a material with outstanding mechanical properties. In contrast to metal valves, there is no plastic deformation, and it is entirely fatigue-free when exposed to prolonged stress.

High leakage occurs especially as a combination of the following four causes:

1. In the pressure-balanced state, there is an initial gap $t_{I}$ of roughly 150 nm between the valve seat and the valve flap due to the manufacturing process (Figure 1a), which cannot be changed easily without accepting multiple different process risks. With applied back pressure, this initial gap between the valve flap and the valve seat starts to close at the front part of the valve flap. With increasing pressure differences, this gap closes further and further until the flap and seat are in contact over the entire flap seat. When the valve is exposed to a very small pressure difference in blocking direction that is not yet sufficient to press the valve flap onto the valve seat (closing the initial gap), the medium can flow through the initial gap in blocking direction.
2. Depending on the valve geometry, pressure differences result in the flap being pressed onto the valve seat, which causes a deformation of the flap. The resulting gaps at the edges of the valve flap, here called remaining gaps, cause leakages. Finite element simulations of this gap between the valve flap and the valve seat are presented in the following.
3. Contaminants, such as particles and fibers, that are located between the valve flap and the valve seat can cause leakage rates and are difficult to avoid.
4. The valve flap and the valve seat are made of silicon and form a hard-hard contact in the closed state. The silicon surface roughness of the contact areas is in the sub-nanometer regime; nevertheless, this contact creates additional gaps that lead to (neglectable small) leakages.

**Figure 4:**
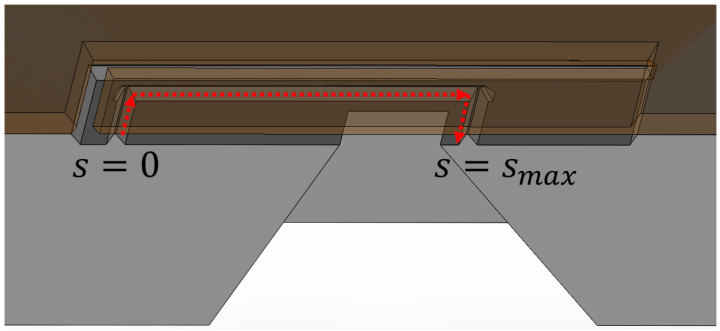
Cross-sectional schematic representation of a half-model showing a transparent valve flap on top of the valve seat. The path along the red arrows is plotted in Figure 5 along the coordinate s as the so-called gap height.

**Figure 5:**
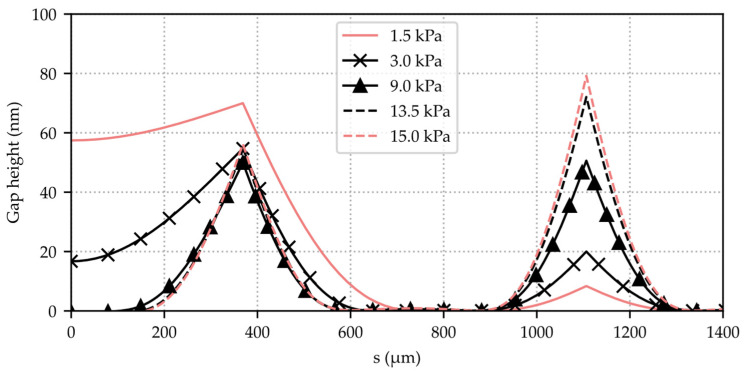
FEM-simulated gap height between the valve seat and the valve flap for several different closing pressures $\Delta p=p_{in}-p_{out}$ plotted against the seat path coordinate s.

Applying pressure against the flap valve opening direction will cause it to bend toward the valve seat until the initial gap of approximately 150 nm is closed. Pushing it even further will now cause duckbill-shaped openings to evolve at the corners as a result of deformations that come along with stresses and bending moments. These have been calculated with steady-state elasto-mechanics and contact-modeling FEM for the representative valve presented in Table 1. The distance between the the valve seat and the bottom of the flap, along the red arrows shown in Figure 4, for several different closing-pressures $p_{in}–p_{out}$ as an outcome of these simulations is plotted over the seat-path distance in Figure 5. Hereby, $p_{in}$ and $p_{out}$ denote the pressure above and below the flap, corresponding to inlet and outlet conditions.

Assuming quasi-static conditions, they represent a channel for undesired backward-leakage flows that need to be predicted, measured, and ideally eliminated. Simply by relating the initial gap to the mean free path of air under atmospheric conditions, one can characterize the flow by Knudsen numbers of O(1), commonly known as Knudsen flow, not being accurately predictable by classic fluid dynamics (continuum) methods. However, the established micropump design methodology heavily relies on them in terms of an efficient and fast optimization, and the adaption process to new applications hence depends on the understanding of these relationships. Utilizing cumbersome Molecular dynamics (MD) or Direct simulation Monte Carlo (DSMC) methods just for leakage mass flow predictions is therefore counterproductive.

Not only continuum modeling, but also conventional experimental methods, reach their limits at very low flow rates. Countermeasures for both deficiencies are presented here, and in connection with this, they are referred to as the extended continuum method. It enables, on the one hand, a fully backwards-compatible and physically sound design flow and, on the other hand, a cost-effective and innovative experimental determination of the smallest gas flows under atmospheric conditions using the pressure decay method, which relates a measured pressure drop within a certain volume to a mass flow rate, as described later.

### 2.3. Comparison of Measurement Procedures for Small Air Flows

The pressure decay measurement is a classic method of industrial leak testing. Nevertheless, it usually lacks detailed evaluation beyond pressure decay times. Typically, a chamber is filled until a certain pressure is reached, and the pressure drop across a given device caused by a leak is measured with a pressure sensor. A quality classification of the tested device by comparing only the pressure drop is possible; nevertheless, in many cases, the volume flow for a quantitative comparison is of interest. This flow can be calculated only with the knowledge of the exact internal volume V and the pressure drop rate Δp/Δt. By accurately determining this internal volume and using a pressure sensor with sufficiently high resolution, this measurement method can be used to calculate low leakage rates, such as those found in silicon microvalves. Alternatively, the necessary gas flow to maintain a certain pressure in the fluidic system can be measured as the leakage rate. In this scenario, the object’s volume needs to be constant, though the exact value can be unknown. While regulating the pressure to a specific level, the flow is monitored with a flow sensor. The expected leakage rates for silicon microvalves at pressure differences < 10 kPa are difficult to measure with standard flow sensors due to limited resolution. For instance, the mass flow meter EL-FLOW Select F-110C from Bronkhorst is suitable for accurate measurement of gas flow ranges between 0.014 cm^3^/min and 0.7 cm^3^/min [8]. Below 0.01 cm^3^/min, the measurement accuracy of classic flow sensors decreases significantly for gases, which leads to increased noise and lower measurement repeatability, whereas pressure can be measured precisely up to the smallest pressure differences.

The capillary method is one more option for the measurement of leakage rates. A capillary with a defined cross-section (e.g., a glass capillary) is used as the fluid line whose leakage current is to be measured. A meniscus is inserted into this capillary, for example, a water meniscus in the case of a gas leak measurement. By observing the meniscus speed (e.g., optically or capacitively), the leakage rate can be determined. However, this method requires manual intervention and can reach its limits at very low flow rates since small capillary pressures are required to set the meniscus in motion. This time-consuming method depends on other parameters, such as the roughness of the inside of the capillary or the evaporation rate of the meniscus, which is superposed on the leakage rate current.

Another leak test procedure is the pressure buildup measurement, where the test pressure is first applied to a volume tank, for example, with an integrated active valve. After reaching the target pressure, an active valve switches and applies the pressure to the device under test (DUT). In the pressurized container connected to the DUT, the pressure increase is measured, which corresponds to the leakage rate of the DUT. This requires additional components, such as a volume tank and an active valve, which are not required for the pressure decay measurement. This means there are additional connections in the pressure buildup measurement from which unwanted leakages can occur. A disadvantage of the described pressure increase or decrease method is the difficulty in deducing the volume flow from the recorded pressure data. Fewer components in the fluidic system are particularly important to reduce the risk of undesired leaks. At the same time, the test bench needs to remain modular to allow easy replacement of the DUT. These advantages, together with the low-cost realization and the possibility to calculate leakage flowrates using a pressure sensor, lead to the decision to realize the concept of a pressure decay measurement test bench.

## 3. Modelling of Knudsen Flows

The aforementioned duckbill gaps (Figure 5) that evolve when the valve flap is pushed against its seat lead to gas flow conditions that cannot be accurately modeled with classical continuum approaches anymore, namely the classical Navier–Stokes equations (CNSE). This condition was originally termed as “Knudsenströmung” (Knudsen flow), characterized by Knudsen numbers above 0.1 (Kn ≥0.1). Lately, a self-diffusion model was derived [9,10,11,12,13] that clearly distinguishes between two different macroscopic flow mechanisms, namely the convective-flux ${\rho \underline{u}}^{C}$ that is driven by external macroscopic forces, as given by, for example, pressure gradients ∇p, and a diffusive flux

(1) $$\dot{{\underline{m}}^{D}}=\rho {\underline{u}}^{D}=-\frac{\mu }{p}\nabla p$$

that is scaled by the molecular viscosity μ and driven by density gradients ∇ρ and/or temperature gradients ∇T within the gas. Under isothermal conditions, the above expression is derived from the kinetic theory of gases and linking the pressure to density by the ideal gas law. The mechanism of Equation (1) is missing in the classical Navier–Stokes equation. Most importantly, by deriving the following relation between the diffusive mass flow rate $M^{D}$ and convective mass flow rate $M^{C}$ through a microchannel, a linkage to the Knudsen number could be established, proving that the lack of the diffusion mechanism within the CNSE is responsible for miss-predicting Knudsen flows [9].

(2) $$\frac{24}{\pi }{Kn}^{2}=\frac{M^{D}}{M^{C}}$$

The key aspect of including self-diffusion into the Navier–Stokes equation is the assumption of a valid superposition of the velocities ${\underline{u}}^{C}$ and ${\underline{u}}^{D}$ in the following manner:

(3) $$\underline{u}={\underline{u}}^{C}+{\underline{u}}^{D}.$$

The stationary continuity equation has to be fulfilled for this total mass flux $\rho \underline{u}$

(4) $$\nabla \cdot \left(\rho \underline{u}\right)=0.$$

In addition, for isothermal stationary gas flows, the following momentum conservation equation has to be fulfilled.

(5) $$\nabla \cdot \left(\rho {\underline{u}}^{C}{\underline{u}}^{C}\right)=-\nabla p-\nabla \cdot {\underline{\underline{\tau }}}^{E},$$

where ${\underline{\underline{\tau }}}^{E}$ represents the extended Newtonian shear-stress tensor.

(6) $${\underline{\underline{\tau }}}^{E}=-\mu \left(\nabla {\underline{u}}^{C}+{\left(\nabla {\underline{u}}^{C}\right)}^{T}\right)+\frac{2}{3}\mu \nabla \cdot {\underline{u}}^{C}\underline{\underline{I}}+\dot{{\underline{m}}^{D}}{\underline{u}}^{C}+{\underline{u}}^{C}\dot{{\underline{m}}^{D}}-\frac{2}{3}\dot{{\underline{m}}^{D}}\cdot {\underline{u}}^{C}\underline{\underline{I}}.$$

Hereby, $\underline{\underline{I}}$ is the unity matrix. The derivation of this tensor based on the kinetic theory of gases is hereby consistent with deriving $\rho {\underline{u}}^{D}$. Often, it is explicitly stated in literature that density and temperature gradients are neglected during this procedure, resulting in only the first three terms, which form the commonly known classical shear-stress tensor. Otherwise, the three additional terms appear, representing the transport of momentum by mass diffusion. Hence, the resulting set of equations (Equation (5)) are called extended Navier–Stokes equations (ENSE) while usually also implicitly including Equations (3) and (4).

### 3.1. Effective Mean Free Path

Based on the kinetic theory of gases, the following expression for the molecular viscosity can be derived:

(7) $$\mu =\frac{1}{3}\rho \lambda \bar{u}\;,$$

where λ is the mean free path of the unconfined medium and $\bar{u}$ is the temperature-dependent mean molecular velocity. It represents the diffusive momentum transport in cross-stream direction and is in the present theory also linked to the streamwise diffusive mass transport. In isothermal cases, it is a constant. However, in this case, no confinement of the medium due to solid boundaries is considered. While this is valid for considerably small Knudsen numbers, for Kn ≥0.01 and especially for molecules near a wall, they cannot travel λ. To take this into account, D. W. Stops [14] proposed the distribution of the actual free path of any gas molecule to obey a probability density function whose actual expression can be established according to the following assumptions:

1. The actual distance traveled by any particle follows an exponential distribution function, as commonly observed in nature.
2. In case of an unconfined medium, the mean free path has to be calculated as λ.

This leads to the distribution function

(8) $$f\left(r\right)=\frac{e^{-\frac{r}{\lambda }}}{\lambda }\;,$$

where r is the distance traveled by any particle between two consecutive collisions. To distinguish the wall-confined mean free path from the unconfined bulk mean free path, the former is referred to as the effective mean free path $\lambda_{eff}$, which can now be calculated as follows in the most general form for a position $\underline{x}$ averaging over spherical coordinates r, ϕ, and θ:

(9) $$\lambda_{eff}\left(\underline{x}\right)=\frac{1}{4\pi }\int_{0}^{2\pi }\int_{0}^{\pi }\left(\int_{0}^{D_{w}\left(\underline{x},\phi ,\theta \right)}rf\left(r\right)dr\;\;\;\;\;\;\;\;\;\;\;\;\;\;\;+D_{w}\left(\underline{x},\phi ,\theta \right)\int_{D_{w}\left(\underline{x},\phi ,\theta \right)}^{\infty }f\left(r\right)dr\right)sin\left(\phi \right)d\phi d\theta .$$

**Figure 6:**
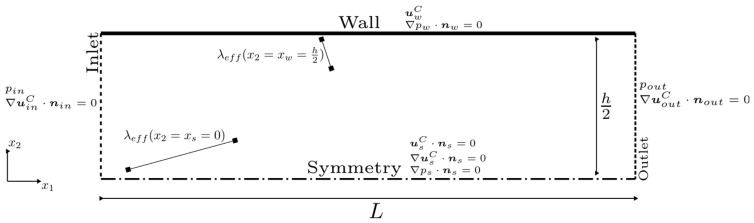
Cross-section and definition of boundary conditions of the simplified microchannel geometry. This section is referring to when explicitly using $x_{2}$ and $x_{1}$ and deriving the analytical solution. $\lambda_{eff}\left(x_{2}\right)$ becomes smaller toward the wall. The subscripts denote any location at the given boundary, whereas $\underline{n}$ represents the surface normal unit vector.

$D_{w}(\underline{x}\;,\phi ,\;\theta ))$ denotes the distance of the particle at a location $\underline{x}$ for a given ϕ and θ to the visible point of the wall boundary. The first term in the brackets is the contribution of particles that travel $0<r<D_{w}$ and the second term of those that travel $r\;∼\;D_{w}$ to the average. Hereby, it is assumed that particles that would collide somewhere behind the wall under unconfined circumstances instead always collide at an infinitesimally small distance $d_{w}\ll \lambda_{eff}\left(x_{w}\right)$ in front of it, where $d_{w}$ is the shortest distance to the wall at $x_{w}$. Due to the promising results of similar approaches [15,16,17], this would be used for evaluating $\lambda_{eff}$ in the present analytical solution for the mass flow. For rectangular gap geometry, a simplified expression for $\lambda_{eff}\left(x_{2}\right)$ can be derived, which is a function of $x_{2}$, as given by [15]. Replacing λ in Equation (7) by $\lambda_{eff}$ leads to an $\mu_{eff}$ to be included in the (Navier–)Stokes framework, as conducted by [16]. Further modeling considerations refer to simplified conditions, as shown in Figure 6.

### 3.2. Wall-Slip Boundary Condition

Wall-slip refers to the existence of a macroscopic convective flux or momentum $\rho {\underline{u}}_{1,w}^{C}$ of the gas medium at a position $x_{w}$ at the wall boundary. This means that, within any distance below a mean free path, no change of the macroscopic gas properties is to be expected, which can be consistently concluded from the derivation of Equation (1) or Equation (7). At this position, the collective of the colliding particles can either carry a momentum corresponding to a convective velocity of:

1. $u_{1,w+}^{C}=u_{1,w}^{C}-\lambda_{eff}\left(x_{w}\right){\frac{\partial u_{1}^{C}}{\partial x_{2}}}_{w}$
2. $u_{1,w,d-}^{C}=v_{1,w}$
3. $u_{1,w,s-}^{C}=u_{1,w}^{C}$

where $v_{1,w}=0$ is the wall’s velocity. In the first case, the particles traveled about a distance of $\lambda_{eff}\left(x_{w}\right)$ toward the wall after their previous collision within the gas, indicated by the subscript +. In case of the two latter $d_{w}$ away from it, whereas in case 2, they have exited the interaction with a wall roughness element corresponding to a diffuse reflection, which is the case if the surface roughness of the wall is considerably larger than the molecular diameter. On the other hand, particles leaving the surface with $u_{1,w,s-}^{C}=u_{1,w}^{C}$ were reflected specularly. For the two latter cases, subscripts d− and s− are used, respectively. Commonly, σ denotes the amount of particles being reflected diffusely. In order for a fulfilled mass balance, the total amount of particles linked to $u_{1,w-}^{C}$:

$$u_{1,w-}^{C}=\sigma u_{1,w,d-}^{C}+\left(1-\sigma \right)u_{1,w,s-}^{C}$$

must be equal to those corresponding to $u_{1,w+}^{C}$. A weighting factor of 0.5 is chosen in order to consider the even contribution of the aforementioned collective of particles to the overall convective flux at the wall:

$$u_{1,w}^{C}=0.5u_{1,w-}^{C}+0.5u_{1,w+}^{C}.$$

From the aforementioned expressions, the following convective velocity at $x_{w}$ with $d_{w}\ll \lambda_{eff}\left(x_{w}\right)$ in front of the wall can be derived [18,19,20,21]:

(10) $$u_{1,w}^{C}=-\frac{2-\sigma }{\sigma }\left(\lambda_{eff}\left(x_{w}\right){\frac{\partial u_{1}^{C}}{\partial x_{2}}}_{w}\right).$$

**Figure 7:**
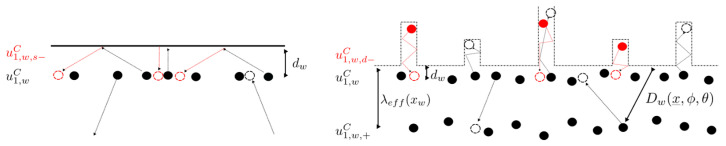
(**Left**) Specular reflection of particles colliding with an ideally smooth wall boundary: Particles keep their tangential momentum: no loss of macroscopic kinetic energy. (**Right**) Diffuse reflection of particles leaving roughness elements of considerably larger size than their diameter: Macroscopic kinetic energy and corresponding tangential momentum of the collective of incident particles is not conserved.

If not explicitly stated otherwise, a fully diffuse reflection σ=1 is assumed in the present work. Figure 7 highlights different types of reflection with respect to the conservation of macroscopic tangential momentum and kinetic energy.

### 3.3. Analytical Solution

It is often desired to model any flow with a minimum amount of complexity for efficient system-level modeling. For the present case, isothermal and stationary conditions have already been introduced also to be consistent with the experimental setup. Even though the flow geometry is slightly more complex, in principle, all assumptions for a rectangular gap flow also hold to be true in this case. Performing a dimensional analysis, the set of momentum, Equation (5), simplifies to the following Stokes flow [19,22,23]:

(11) $$\frac{\partial p}{\partial x_{1}}=\mu_{eff}\frac{\partial }{\partial x_{2}}\left(\frac{\partial u_{1}^{C}}{\partial x_{2}}\right)\;\frac{\partial p}{\partial x_{2}}=0.\;\;\;\;\;$$

Given the boundary condition at the wall at Equation (10), the following expression for $u_{1}^{C}$ can be derived:

(12) $$u_{1}^{C}=\frac{1}{2\mu_{eff}}\frac{\partial p}{\partial x_{1}}\left(x_{2}^{2}-\frac{h^{2}}{4}-\frac{2-\sigma }{\sigma }\lambda_{eff}\left(x_{w}\right)h\right).$$

Considering Equation (3), the total velocity $u_{1}$ is calculated as follows:

(13) $$u_{1}=u_{1}^{C}+u_{1}^{D}=\frac{1}{2\mu_{eff}}\frac{\partial p}{\partial x_{1}}\left(x_{2}^{2}-\frac{h^{2}}{4}-\frac{2-\sigma }{\sigma }\lambda_{eff}\left(x_{w}\right)h\right)-\frac{\mu_{eff}}{\rho p}\frac{\partial p}{\partial x_{1}}.$$

By multiplying this expression with ρ, performing integration over $x_{2}$, $x_{3}$ and averaging over $x_{1}$, the total mass flow $\dot{M}$ can be derived as [9,18,19,21,22].

(14) $$\dot{M}\left(h\left(x_{3}\right),w,L,p_{in},p_{out},R,T\right)\;\;\;\;\;\;\;\;\;\;\;\;\;\;\;\;\;\;\;=\int_{0}^{w}\int_{0}^{\frac{h\left(x_{3}\right)}{2}}\left(\left(\frac{h^{2}}{8\mu_{eff}\left(x_{2}\right)}-\frac{x_{2}^{2}}{2\mu_{eff}\left(x_{2}\right)}\;\;\;\;\;\;+\frac{2-\sigma }{\sigma }\frac{h\lambda_{eff}\left(x_{w}\right)}{2\mu_{eff}\left(x_{2}\right)}\right)\frac{\left(p_{in}^{2}-p_{out}^{2}\right)}{RTL}\;\;\;\;\;+\frac{2\mu_{eff}\left(x_{2}\right)}{L}ln\left(\frac{p_{in}}{p_{out}}\right)\right)dx_{2}dx_{3}$$

As given by Equation (4), the total mass flux $\dot{M}$ is constant at each $x_{1}$ of the microchannel. L represents the channel length, w the width, $h\left(x_{3}\right)$ the height at $x_{3},$ and R the specific gas constant. Equation (14) is used as the final result for the following method on the analytical prediction of Knudsen flows at silicon flap valves, where they are considered as leakages, modeled in the general extended Navier–Stokes (ENSE) form Equations (1) and (3)–(6).

### 3.4. Model Validation

**Figure 8:**
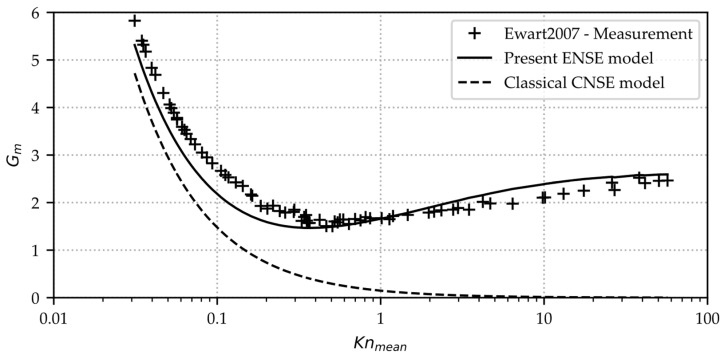
Non-dimensional mass flow plotted against the mean Knudsen number. Comparison of present extended modeling approach mass flow Equation (14), classical Navier–Stokes solution, and experimental data of [18].

The mass flow results obtained by Equation (14) are compared with the subset of the experimental data presented in [18] for a steady helium flow ($R=\frac{R_{uni}}{M_{hel}},\;\mu =1.97513\;\mathrm{Pa}\;s$), specified by an inlet/outlet pressure ratio of $\frac{p_{in}}{p_{out}}=5$, through a rectangular microchannel with channel dimensions of w=492  μm, L=9.39 mm, and h=9.38 μm by plotting the non-dimensional mass flow $G_{m}$

(15) $$G_{m}=\dot{M}{\left(\frac{wh^{2}}{L\sqrt{2RT}}\left(p_{in}-p_{out}\right)\right)}^{-1}\;$$

against the mean Knudsen number $Kn_{m}$

(16) $$Kn_{mean}=\frac{\sqrt{\pi }}{2}\frac{\mu }{0.5\left(p_{in}+p_{out}\right)}\sqrt{2RT}\;$$

as shown in Figure 8.

As the surface roughness is given to be <20 nm, a fully diffuse reflection is hereby assumed σ=1. The calculated results therefore represent lower bounds in terms of the actual mass flow rate. As far as the present work investigations go, for this set of experimental data, the results of the present extended Navier–Stokes model show sufficient validity. The deviation stays below 25% for each set of ($G_{m}$, $Kn_{mean}$). The experimental methodology applied there to obtain the mass flow rates from measured pressure values is similar to the present case explained later. The main difference is that the Knudsen numbers are achieved by coarse vacuum (inlet and outlet) with 10 µm height channel made of silicon rather than sub-micrometer channel heights under atmospheric conditions, as in the present case. This could affect the gas-surface interaction in terms of σ differently. The uncertainty for the mass flow is given as 5%. Results of similar experiments with different gases are not expected to deviate drastically when normalized accordingly [24].

## 4. System Design and Data Processing

**Figure 9:**
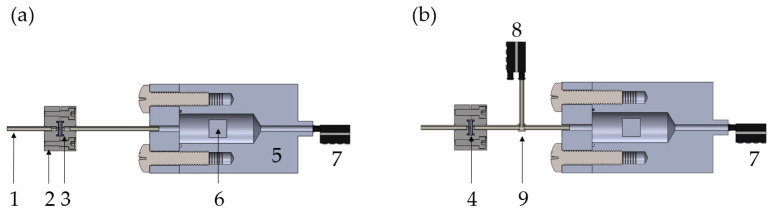
Schematic cross-section through CAD model of the developed pressure decay system (**a**,**b**). (1) Capillary, (2) aluminum valve housing, (3) silicone sealing, (4) microvalve (DUT), (5) aluminum pressure chamber, (6) removable calibration test weight, (7) pressure sensor 1 (HSC-DRRN600MD2A3), (8) pressure sensor 2 (HSC-DRRN160MD2A5) only in (**b**), and (9) capillary T-connector.

This section details the developed pressure decay test bench with two different setups in detail. Figure 9 depicts the schematic cross-section through the computer-aided design (CAD) of the setup (SolidWorks 2022). PEEK capillaries (1) fluidically connect the system, and an aluminum housing (2) is designed to fit the silicon microvalve (4). To prevent leakages, a silicone sealing (3) is inserted between the silicon valve and the aluminum housing. The aluminum pressure chamber (5) with the removable calibration test weight (6) is connected to one pressure sensor (7). Another pressure sensor (8) with a smaller measuring range is joined with a capillary T-connector (9) only in Figure 9b. This second pressure sensor is optional and only included for validation purposes in Section 5.2, nevertheless adapting the pressure range with multiple pressure sensors is possible and gives the system flexibility for a wide variety of measuring ranges.

**Figure 10:**
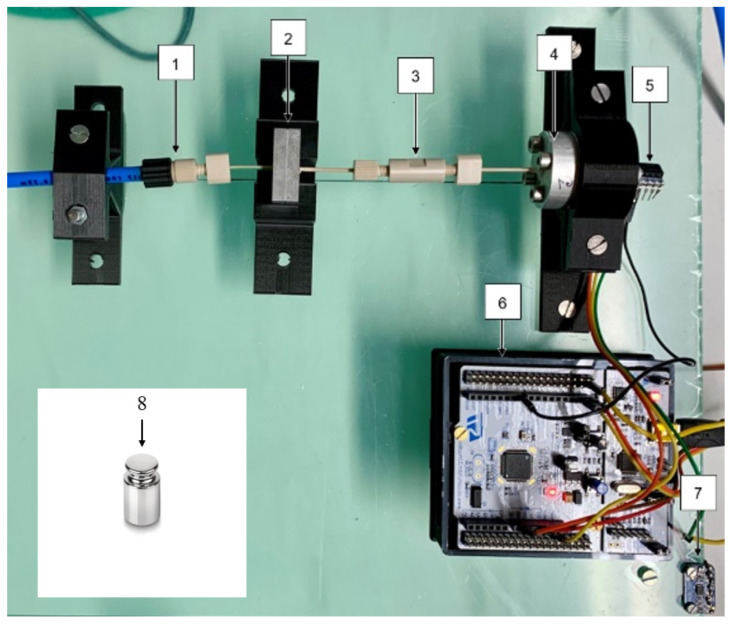
Pressure decay system setup shown schematically in Figure 9a with one connected pressure sensor: (1) fluidic connection to pressure generator CPC4000, (2) aluminum housing for silicon microvalve, (3) capillary with connector, (4) pressure chamber, (5) pressure sensor HSC-DRRN600MD2A3, (6) NUCLEOF411RE microcontroller, (7) MCP9808 temperature sensor, and (8) calibration test weight.

The realized test bench, depicted in Figure 10, includes a pressure generator CPC4000, which is connected to the test valve or micropump housing (2) via a capillary (1). To prevent leakages around the DUT, an additional silicone sealing gasket is inserted into the housing. This sealing was tested with a blank silicon chip without a valve to ensure the tightness of the sealing. After several minutes, no pressure loss is detected; hence, the overall system is considered free of leakages.

The DUT can be replaced easily, quickly, and non-destructively. A capillary (3) connects the housing with a pressure chamber (4) for pressure buildup and can be disassembled in order to insert the calibration test weight (8) for a defined volume reduction. Attached to the test bench is a temperature sensor MCP9808 (7) to record temperature fluctuations of the ambient air for the calculation of the leakage rates. The ambient air temperature is assumed to be the gas temperature of the leakage flow. Sensor data is processed by an STM microcontroller NUCLEO411RE (6) and transferred to a computer for processing.

### Data Processing and Flow Calculation

**Figure 11:**
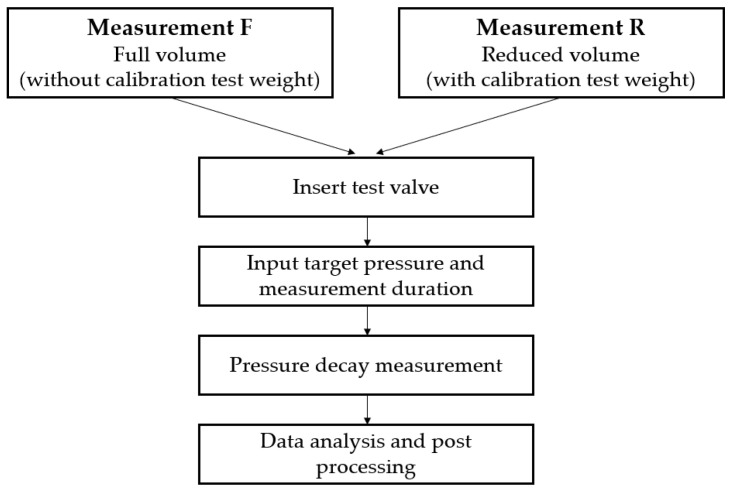
Simplified flow diagram of testing procedure. Two measurements without defined volume reduction (Measurement F) and with defined volume reduction (Measurement R) due to an inserted calibration test weight are used for the determination of the volume of the pressure chamber and periphery.

A 1 g steel calibration test weight, depicted in Figure 10 (8), as a defined volume for a defined volume reduction of the pressure chamber is used to determine the internal volume of the pressure chamber and periphery, such as capillaries and connectors. We assume that the calibration test weight has a precisely determinable volume, which can be calculated based on temperature T=20 °C, density ρ=7859 kg/m^3^, and weight $m_{1g}=1\times 10^{-3}$ kg, verified with a laboratory balance, results in $V_{D}=V_{1g}=1.272\times 10^{-7}$ m^3^. The described test procedure is depicted in Figure 11.

**Figure 12:**
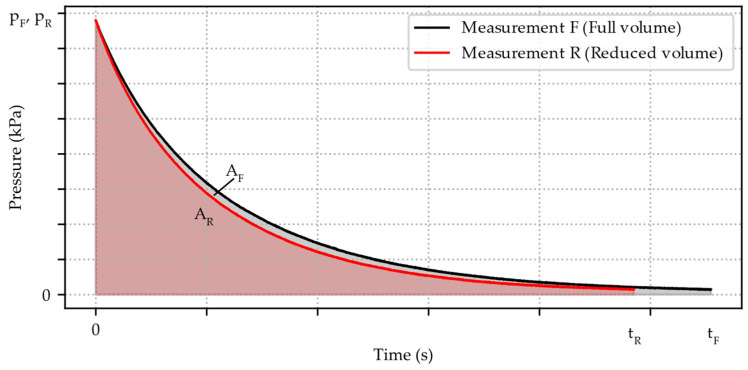
Two pressure decay measurements schematically displayed, performed with full volume and no inserted calibration weight (Measurement F) and with reduced volume with inserted calibration weight (Measurement R). Starting pressures $p_{R}(t=0\;s)$ and $p_{F}(t=0\;s)$, atmospheric pressure at $t_{F}$ and $t_{R}$. Integrated area below black graph $\left(A_{F}\right)$ and below red graph $\left(A_{R}\right)$ used for the internal volume calculation.

Two consecutive pressure decay measurements are necessary to compute the internal volume of the pressure chamber and periphery, as depicted in Figure 12. After determining the internal volume, it is no longer necessary to carry out this measurement procedure of two consecutive pressure decay measurements. Therefore, one measurement with the known internal volume of the system is sufficient, if this internal volume is not modified by changing the specimen.

One measurement with inserted test volume for the defined volume reduction in the pressure chamber (Figure 12, Measurement R) as well as one measurement without inserted test volume is performed (Figure 12, Measurement F). The temperature is constantly measured and observed in the controlled lab environment. All necessary steps to calculate the leakage volume flow from those pressure decay measurements are explained in the following. The ideal gas equation

(17) $$n\left(t\right)=p\left(t\right)\frac{V}{R\cdot T}$$

depicts the substance amount n of air in relation to the volume V and the ideal gas constant of air R. Assuming a constant temperature T, the substance amount n in the volume V at a certain similar pressure level p is obtained. The ratio r (at the same temperature T and pressure p) can be compared and assumed to be equal.

(18) $$r\left(p\right)=\frac{n_{F}(t_{F})}{V_{F}}=\frac{n_{R}(t_{R})}{V_{R}}$$

By integrating over the measurement time t until atmosphere pressure is reached from the beginning of the pressure decline, the area under the curve is obtained and gives a relation to the volume V of the pressure chamber and periphery.

(19) $$\int_{0}^{t_{F}}p_{F}\left(t\right)dt=\frac{V_{F}}{V_{R}}\int_{0}^{t_{R}}p_{R}\left(t\right)dt$$

$A_{F}$ and $A_{R}$ represent the integrated area under measurement F or measurement R. The ratio between $A_{F}$ and $A_{R}$ is assumed similar to the ratio between volume $V_{F}$ and volume $V_{R}$. The combined heuristic formula used for the volume determination is obtained.

(20) $$A_{F}=\int_{0}^{t_{F}}p_{F}\left(t\right)dt=\frac{V_{F}}{V_{R}}\int_{0}^{t_{R}}p_{R}\left(t\right)dt=\frac{V_{F}}{V_{R}}\cdot A_{R}$$

As explained above, measurement R has a defined reduced volume $V_{D}$ due to the inserted calibration weight, which defines the test volume. To compute our unknown chamber volumes $V_{F}$ and $V_{R}$ from which the pressure decrease is measured, $V_{R}$ gets replaced with ${V_{F}-V}_{D}$.

(21) $$\frac{A_{R}}{A_{F}}=\frac{V_{R}}{V_{F}}=\frac{V_{F}-V_{D}}{V_{F}}$$

With Equation (21), the unknown volumes $V_{F}$ and $V_{R}$ are calculated, as seen in Formulas (22) and (23).

(22) $$V_{F}=V_{D}\frac{A_{F}}{A_{F}-A_{R}}$$

(23) $$V_{R}=V_{D}\frac{A_{R}}{A_{F}-A_{R}}$$

Assuming an ideal gas and using the calculated volume of the pressure chamber V to receive the substance amount Δn for a measured pressure drop Δp.

(24) $$\Delta n=\frac{-\Delta p\;\cdot \;V}{R\;\cdot \;T}$$

The obtained amount of substance Δn leads to the mass flow $q_{m}$ by dividing the latter-defined time step Δt and multiplying it with the molar mass M of atmospheric air.

(25) $$q_{m}(t)=\frac{\Delta n\;\cdot \;M}{\Delta t}$$

Necessary for determining the volume flow $q_{v}(t)$ is the pressure-dependent fluid density of air ρ(t).

(26) $$\rho (t)=\frac{p(t)\;\cdot \;M}{R\;\cdot \;T}\;$$

From the obtained mass flow $q_{m}(t)$, the volume flow $q_{v}(t)$ can be determined by considering the fluid density of air ρ(t).

(27) $$q_{v}(t)=\frac{q_{m}(t)}{\rho (t)}\;$$

## 5. Results

This section shows results of exemplary measurements and simulations for gas leakage rates. Multiple different silicon microvalve samples with identical geometries were used and evaluated with the developed test bench. Those silicon microvalves were manufactured in the cleanroom facility at Fraunhofer EMFT in Munich.

### 5.1. Measurement Results

**Figure 13:**
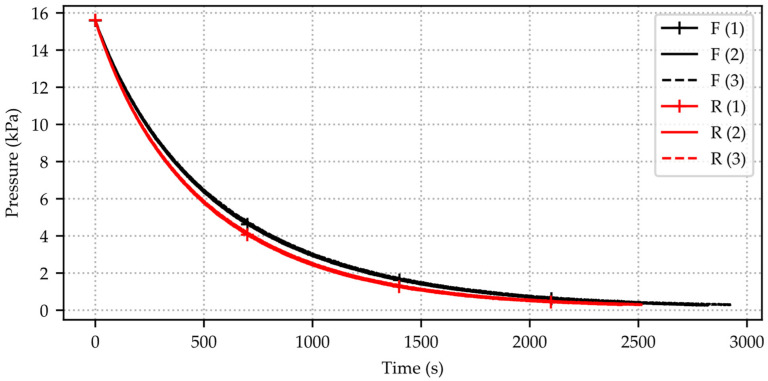
Six air pressure decay measurements with the same silicon microvalve sample µV 06 C1206. Three consecutive measurements with inserted calibration test weight $V_{D}=V_{1g}=1.272{\;\times \;10}^{-7}$ m^3^ or $m_{1g}=1{\;\times \;10}^{-3}$ kg (R = reduced volume) and three consecutive measurements without calibration test weight (F = full volume). The plot is generated with time series data from pressure sensor 2 (Honeywell HSC-DRRN160MD2A5).

In the following subsection, results obtained with the pressure decay measurement method are presented. The repeatability of this method is demonstrated with one housed silicon microvalve sample (µV 06 C1206) from Fraunhofer EMFT. In total, six consecutive pressure decay measurements are carried out, along with three measurements with inserted calibration test weight (R = reduced volume) and three measurements without inserted calibration test weight (F = full volume). The result in each case is a time-decreasing function of pressure over time, depicted in Figure 13.

**Figure 14:**
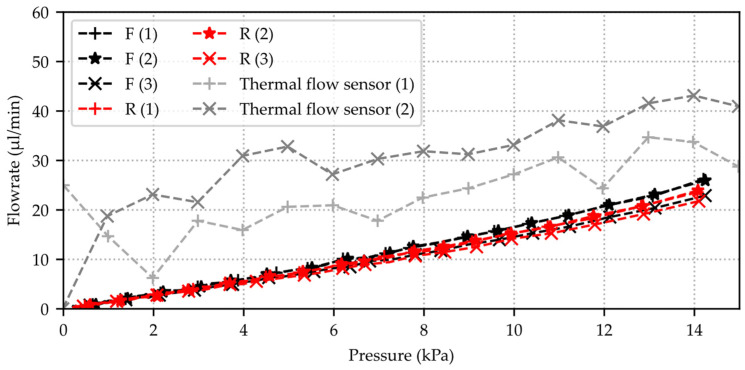
Calculated air leakage volume flows for the corresponding measurements from Figure 13 with the same silicon microvalve sample µV 06 (C1206) after internal volume calculation (Table 2). Measurements with inserted calibration weight (R = reduced volume) and without inserted calibration weights (F = full volume) are depicted, and the air leakage flow rate was obtained with every 600th datapoint (slice 600) with time series data from pressure sensor 2 (Honeywell HSC-DRRN160MD2A5). Two consecutive reference measurements with microvalve sample performed with thermal mass flow sensor EL-FLOW Select F-110C from Bronkhorst.

These measurements are used to determine the internal volume of the system from which the pressure is released. Prior to each measurement, the entire fluidic system was dismantled and reassembled, and an overpressure of 15 kPa relative to the atmospheric pressure is generated inside the pressure chamber. Due to the leakage rate of the microvalve and periphery, this overpressure equalizes to atmospheric pressure after 2000–2800 s. This sample with higher leakage volume flow is selected for the volume determination as the measurement is performed in a rapid manner minimizing the risk of temperature fluctuations as well as being able to validate the data with a state-of-the-art mass flow sensor (Figure 14).

The volume flow of this silicon microvalve µV 06 C1206 is determined from the pressure decay data using the methodology described in Section 3.3. Each of the three measurements with included test weight (reduced volume), shown in Figure 13, is compared to the corresponding measurement without test weight (full volume). Calculated leakage flow starts from ~14 kPa relative to atmospheric pressure due to the data slice in order to eliminate noise from the data. The internal volume of the pressure chamber (without test volume) is determined each for the two corresponding measurements and shown in Table 2. The calculated mean volume $\bar{V}$ as a result of all three measurements and the percentage difference from the mean $\left|V_{i}-\bar{V}\right|$ are depicted. The percentage deviation from the mean volume is below 7% over all three measurements with pressure sensor 1 and below 4.5% for pressure sensor 2. The total error band of the used pressure sensors is 1% and can therefore be considered as the minimum error of this measurement method. Additional error sources that can negatively influence the results are temperature fluctuations, possible leaks in the system, and particles.

Figure 14 depicts the obtained leakage volume flow with the pressure decay system after determining the internal volume of the system. Six consecutive measurements with valve µV 06 C1206 are superimposed and indicate almost-identical results with increasing deviation at higher pressures. There is no recognizable difference in the flow rate whether the internal volume is reduced by the inserted test volume. The measured flow rate with the state-of-the-art thermal mass flow sensor shows an increased noise and higher absolute values for the flow rate compared to the pressure decay method, although the gradient is in the same range. The comparison of both measurement methods is carried out on this valve, as the leakage rate is still partly within the measuring range of the thermal mass flow sensor.

**Figure 15:**
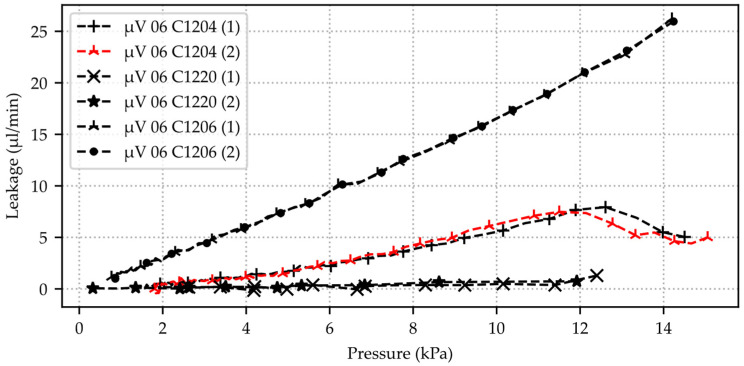
Calculated air leakage volume flow for three silicon microvalves of type µV 06. Each valve is measured twice with the pressure decay method starting from 13–15 kPa air overpressure relative to atmospheric pressure until near atmospheric pressure. Time series data from pressure sensor 2 is used for calculation (Honeywell HSC-DRRN160MD2A5).

After demonstrating the reproducibility of the measurement method using several consecutive measurements with one sample, additional silicon microvalves with the same design (µV 06), as detailed in Table 1, are used for leakage flow measurements. Figure 15 illustrates the calculated air leakage rate of three samples from valve type µV 06 based on two consecutive pressure decay measurements each. The maximum volume flow obtained is ~26 µL/min air at a pressure difference of 14 kPa relative to atmospheric pressure with valve C1206. Valve C1220 shows air flow rates in blocking direction of 1–2 µL/min at 11–12 kPa. The starting pressure varies due to different measurement durations of the valves and the associated different number of averaged values. Every ${1\times 10}^{4}$th pressure sensor value (slice 10,000) is recognized for the computation and plotting of valve C1204, slice 300,000 for C1220, and slice 600 for C1206. Both consecutive measurements of the same valve show high reproducibility. However, the absolute leakage volume flow varies considerably across the different valve samples. The trend at smaller pressure differences is almost linear in the direction of atmospheric pressure.

### 5.2. Comparison between Modeling and Measurement of Air Leakage Rates

**Figure 16:**
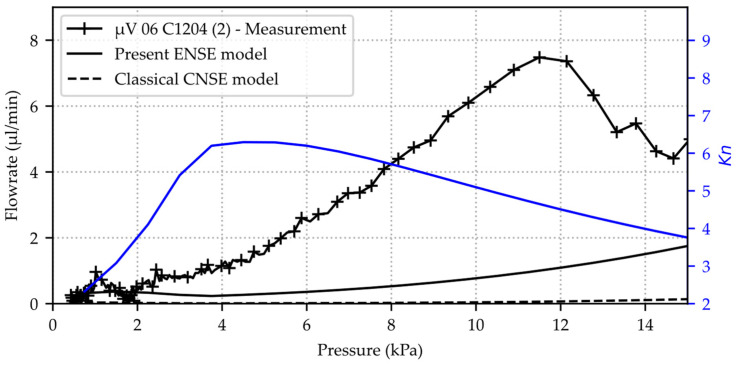
Comparison of the present experimental data of sample µV 06 C1204 (marked red in Figure 15) with the predicted values as given by Equation (28) of the volume flow rate plotted over $\Delta p=p_{in}-p_{out}$. Volume flow weighted average (theory) of the Knudsen number (blue curve) is additionally shown to provide information about the actual flow conditions.

The flux-weighted average of the Knudsen number is plotted in Figure 16 against $\Delta p=p_{in}-p_{out}$ together with the theoretical total volume-flow rate

(28) $${\dot{V}}_{Leakage}\left(p_{in}\right)=\frac{\dot{M}(h_{Gap},\;w_{Max},L_{Gap},p_{in},p_{out},R_{Air},T)}{\rho_{Air}}\;,$$

where the latter is evaluated by using the gap height $h_{Gap\;}\left(s=x_{3},\Delta p\right),$ as plotted in Figure 5, together with the corresponding $w_{max}=s_{Max}$ as integration limits within $\dot{M}$, as given by Equation (15). The valve’s seat length $L_{Gap}=w_{s}$ is printed in Table 1. In order to quantify the impact of the assumptions for deriving the analytical solution Equation (15) and its application on the gap flow geometries in the presented way, the set of equations Equations (4) and (5) is solved for ${\underline{u}}^{C}$ and p by the OpenFOAM ENSE-solver presented in [7], where wall-slip ${\underline{u}}_{w}^{C}\;\neq \;0$ and $\mu_{eff}$ are not considered at this point. Hereby, the actual gap geometry is converted from the elasto-mechanics FEM export into the corresponding polyLine format of the CFD meshing tool blockMesh, in whose generated output they are treated as walls by the solver. Upon convergence, the total mass flow rate is derived from the solution variables by Equation (3), and the relative deviation to Equation (15) is then calculated. For several different sets of $p_{in}$ and $h\left(s\right)$, the latter falls between 10% and 20%.

Additionally, the experimental data of sample µV 06 C1204 (marked red in Figure 15) is plotted in Figure 16 for comparison. This sample is selected since the measured leakage flow is in the mid-range of the three measured valves. As expected, the CNSE solution is almost neglectably low and barely fits any of the measurements, while the present extended model is somewhat closer but, depending on the valve sample, still significantly off. Yet, a deviation below two orders of magnitude could be achieved.

## 6. Discussion

The pressure decay method enables the quantification of the smallest gas flows for various devices, presented in this publication for gas leakage rates in blocking direction of silicon microvalves. Nevertheless, this measurement has some drawbacks: A measurement may take several days, depending on the chamber volume in which the pressure is built up and the resulting volume flow. This is a disadvantage and severely limits the possibility of carrying out a large number of measurements. Temperature variations in the laboratory have a major impact on the pressure in the system and, therefore, the result and stability of the measurement, so the measurement would have to be performed in a climatic chamber to improve the results. These errors can be reduced by analyzing the measured values by averaging (slicing) them. This reduces the number of points at which the flow is evaluated and decreases the noise, and it is performed in the measurements presented above. By selecting different pressure sensors depending on the expected flow rates, we have been able to show that the noise can be reduced and the accuracy of the measurement increased. This allows precise measurements to be carried out in a wide variety of measuring ranges, demonstrated in this case on a sample with very low gas volume flows <20 µL/min. The three measured specimens scatter considerably, and the reasons for this are only suspected, such as particles, manufacturing tolerances, defects in the valve or housing, and so forth. As the flow conditions are characterized with large Knudsen numbers due to the small leakage gaps, a fully consistent extended Navier–Stokes model for Knudsen flow (Kn>1) is presented as a slightly complemented consolidation of past works. This was substantiated by validating the model with the renowned experimental data of [18] for mean Knudsen numbers up until 50. However, considering the emphasized model validity and the scattered experimental data, the comparison with the experimental data of one sample in Figure 16 indicates very likely that neither of those deviations is due to modeling errors. However, if a partly specular reflection is assumed, higher flow rates are calculated. The surface roughness of the valves seat is around 0.3 nm and therefore in the same order of magnitude as the van der Waals radius of nitrogen. Therefore, the value for σ might be considerably below 1; a value of σ=0.2 [25] was one of the lowest reported in literature. Applying a simple arithmetic expression to calculate a quantity of interest is generally the most desired and efficient way, as it is also for the leakage estimation in the form of the analytical solution Equation (15) in present work. It can be derived by making assumptions that hold true for the majority of steady and isothermal microchannel flow situations with infinitely extended plane-parallel plate geometries. However, in principle, they also apply for the present leakage gap flow boundaries, which have a slightly increased complexity. A comparison of the analytical solution Equation (15) with the CFD results shows a deviation below 20%, quantifying the viability of applying the former equation respective to the presented methodology. However, wall-slip ${\underline{u}}_{w}^{C}\;\neq \;0$ and $\mu_{eff\;}$ is not yet considered.

## 7. Conclusions

This publication shows a straightforward and cost-effective test bench for the precise determination of the smallest gas flows, even far below 0.1 cm^3^/min using the pressure decay method. In this process, flow rates below the resolution limit of flow sensors can be measured reliably and reproducibly, shown exemplarily with three silicon microvalves of the same design. Nevertheless, a climatic chamber with a constant temperature is recommended for these time-consuming measurements to reduce noise in the leakage volume flow. For measurements on samples with lower expected flow rates, an alternative pressure sensor with a reduced operating range was successfully integrated into the system, and a reduced noise level was detected in the data. This allows the detection of leakage volume flows with higher resolution and shows the system adaption capabilities for different pressure and leakage ranges. The accurate calculation of the test volume with data from both pressure sensors is elaborate and ensures reproducible measurements. The empirical data provide a robust foundation and allow a comparison between the measurement data and the theoretical model. The latter being termed as extended Navier–Stokes (ENSE) model as presented in this work is expected to predict gas flows for Knudsen numbers of up to 50, as shown in the validation section. The presented ENSE variant includes not only self-diffusion but also wall-slip and an effective mean free path model. However, the different measured samples in the present work deviate from this model’s prediction for a maximum factor of 10. The precise value of σ has to be clarified either way. Wall-slip ${\underline{u}}_{w}^{C}\;\neq \;0$ and $\mu_{eff}$ for arbitrary complex geometries could be furthermore implemented into the OpenFOAM ENSE-solver in order to stretch the model predictions to the maximum accuracy. With respect to the observed scattered experimental values, their deviation from the model predictions and the comparably low deviation between CFD and analytics (below 20%) without these implementations is not considered as the first priority to look up into. Further steps will include extensive measurements with changing specimens, and leakage improved passive silicon microvalves and micropumps in order to develop micropumps for different applications with challenging leakage requirements.

## Figures and Tables

**Table 1:** Designed geometric parameters of the valve and values, including flap length l, flap width b, flap thickness $t_{v}$, inlet opening length and inlet opening width $w_{I}$, length from flap suspension to the beginning of acting inlet opening pressure *α*, length from flap suspension to the end of acting inlet opening pressure *β*, KOH angle γ, and valve seating width $w_{S}$.

Valve Type	Symbol	Value	Unit
µV 06	l	1099	µm
	b	802	µm
	$t_{v}$	14	µm
	$w_{I}$	732	µm
	$t_{I}$	150	nm
	A	339	µm
	Β	1071	µm
	$w_{S}$	2.7	µm

**Table 2:** Resulting internal chamber volumes from measurements depicted in Figure 13. Full chamber volumes $V_{F}$ without inserted test volume $V_{D}$, mean chamber volume $\bar{V,}$ and percentage deviation from the mean chamber volume. Calculation performed with pressure sensor 1 (Honeywell HSC-DRRN600MD2A3) and pressure sensor 2 (Honeywell HSC-DRRN160MD2A5) for comparison.

	Measurement		
**Pressure Sensor 1**	**1**	**2**	**3**
$\mathrm{Full}\;\mathrm{Volume}\;V_{F}$ (cm^3^)	1.142	1.227	1.078
$\bar{V}$ (cm^3^)		1.149	
$\left|V_{i}-\bar{V}\right|$ (%)	0.61	6.79	6.18
**Pressure Sensor 2**	**1**	**2**	**3**
$\mathrm{Full}\;\mathrm{Volume}\;V_{F}$ (cm^3^)	1.158	1.182	1.096
$\bar{V}$ (cm^3^)		1.145	
$\left|V_{i}-\bar{V}\right|$ (%)	1.11	3.20	4.31

## Data Availability

Dataset available on request from the authors.

## References

[1] Oh K.W., Ahn C.H. (2006). A review of microvalves. J. Micromech. Microeng..

[2] Bußmann A.B., Grünerbel L.M., Durasiewicz C.P., Thalhofer T.A., Wille A., Richter M. (2021). Microdosing for drug delivery application—A review. Sens. Actuators A Phys..

[3] Leistner H., Wackerle M., Congar Y., Anheuer D., Roehl S., Richter M., Schlaak H. (2021). Robust Silicon Micropump of Chip Size 5 × 5 × 0.6 mm^3^ with 4 ml/min Air and 0.5 ml/min Water Flow Rate for Medical and Consumer Applications. Proceedings of the Actuator 2021, International Conference and Exhibition on New Actuator Systems and Applications: GMM Conference, Online Event, 17–19 February 2021.

[4] Richter M., Anheuer D., Wille A., Congar Y., Wackerle M. (2023). Multistage Micropump System towards Vacuum Pressure. Actuators.

[5] Lewis R., Coolidge C.J., Schroeder P.J., Bright V.M., Lee Y.C. (2013). Fabrication, assembly, and testing of a MEMS-enabled micro gas compressor for a 4: 1 pressure ratio. Sens. Actuators A Phys..

[6] Durasiewicz C.P., Güntner S.T., Maier P.K., Hölzl W., Schrag G. (2021). Piezoelectric Normally Open Microvalve with Multiple Valve Seat Trenches for Medical Applications. Appl. Sci..

[7] Schwarz J., Axelsson K., Anheuer D., Richter M., Adam J., Heinrich M., Schwarze R. (2023). An OpenFOAM solver for the extended Navier–Stokes equations. SoftwareX.

[8] Bronkhorst Deutschland Nord Gmbh Datasheet F-110C: EL-FLOW Select F-110C. https://www.bronkhorst.com/de-de/produkte/gas-durchfluss/el-flow-select/f-110c/.

[9] Sambasivam R. (2012). Extended Navier-Stokes Equations: Derivations and Applications to Fluid Flow Problems. Ph.D. Dissertation.

[10] Chakraborty S., Durst F. (2007). Derivations of extended Navier-Stokes equations from upscaled molecular transport considerations for compressible ideal gas flows: Towards extended constitutive forms. Phys. Fluids.

[11] Dongari N., Sambasivam R., Durst F. (2009). Extended Navier-Stokes Equations and Treatments of Micro-Channel Gas Flows. J. Fluid Sci. Technol..

[12] Dongari N., Sharma A., Durst F. (2009). Pressure-driven diffusive gas flows in micro-channels: From the Knudsen to the continuum regimes. Microfluid. Nanofluidics.

[13] Durst F., Gomes J.P., Sambasivam R. Thermofluiddynamics: Do We Solve the Right Kind of Equations? In Turbulence Heat and Mass Transfer 5. Proceedings of the International Symposium on Turbulence Heat and Mass Transfer.

[14] Stops D. (1970). The mean free path of gas molecules in the transition regime. J. Phys. D Appl. Phys..

[15] Arlemark E.J., Dadzie S.K., Reese J.M. (2010). An Extension to the Navier–Stokes Equations to Incorporate Gas Molecular Collisions With Boundaries. J. Heat Transf..

[16] Guo Z.L., Shi B.C., Zheng C.G. (2007). An extended Navier-Stokes formulation for gas flows in the Knudsen layer near a wall. Europhys. Lett..

[17] Dongari N., Durst F., Chakraborty S. (2010). Predicting microscale gas flows and rarefaction effects through extended Navier–Stokes–Fourier equations from phoretic transport considerations. Microfluid. Nanofluidics.

[18] Ewart T., Perrier P., Graur I.A., Méolans J.G. (2007). Mass flow rate measurements in a microchannel, from hydrodynamic to near free molecular regimes. J. Fluid Mech..

[19] Maurer J., Tabeling P., Joseph P., Willaime H. (2003). Second-order slip laws in microchannels for helium and nitrogen. Phys. Fluids.

[20] Maxwell J.C. (1879). VII. On stresses in rarified gases arising from inequalities of temperature. Philos. Trans. R. Soc. Lond..

[21] Dong J.-W., Huang C.-Y. (2023). A comprehensive non-kinetic approach for rarefied gas flow between parallel plates. Phys. Fluids.

[22] Tomy A.M., Dadzie S.K. (2022). Diffusion-Slip Boundary Conditions for Isothermal Flows in Micro- and Nano-Channels. Micromachines.

[23] Durst F., Filimonov D., Sambasivam R. (2020). Treatments of Micro-channel Flows Revisited: Continuum Versus Rarified Gas Considerations. J. Inst. Eng. Ser. C.

[24] Hemadri V., Varade V.V., Agrawal A., Bhandarkar U.V. (2016). Investigation of rarefied gas flow in microchannels of non-uniform cross section. Phys. Fluids.

[25] Agrawal A., Prabhu S.V. (2008). Survey on measurement of tangential momentum accommodation coefficient. J. Vac. Sci. Technol. A Vac. Surf. Film..

